# Structure, Bioactivity and Analytical Methods for the Determination of *Yucca* Saponins

**DOI:** 10.3390/molecules26175251

**Published:** 2021-08-30

**Authors:** Gabriel G. Jiménez, Alexandra G. Durán, Francisco A. Macías, Ana M. Simonet

**Affiliations:** Allelopathy Group, Department of Organic Chemistry, Campus de Excelencia Internacional (ceiA3), Institute of Biomolecules (INBIO), School of Science, University of Cadiz, c/República Saharaui 7, 11510 Puerto Real, Spain; gabriel.garciajimenez@alum.uca.es (G.G.J.); alexandra.garcia@uca.es (A.G.D.)

**Keywords:** saponin, *Yucca*, steroidal, spirostanic glycoside, bioactivity, analytical methods

## Abstract

*Yucca* is one of the main sources of steroidal saponins, hence different extracts are commercialized for use as surfactant additives by beverage, animal feed, cosmetics or agricultural products. For a deeper understanding of the potential of the saponins that can be found in this genus, an exhaustive review of the structural characteristics, bioactivities and analytical methods that can be used with these compounds has been carried out, since there are no recent reviews on the matter. Thus, a total of 108 saponins from eight species of the genus *Yucca* have been described. Out of these, the bioactivity of 68 saponins derived from the isolation of *Yucca* or other genera has been evaluated. Regarding the evaluation and quality control of the saponins from this genus LC-MS technique is the most often used. Nevertheless, the development of methods for their routine analysis in commercial preparations are needed. Moreover, most of the studies found in the literature have been carried out on *Y. schidigera* extract, since is the most often used for commercial purposes. Only eight of the 50 species that belong to this genus have been studied, which clearly indicates that the identification of saponins present in *Yucca* genus is still an unresolved question.

## 1. Introduction

The *Yucca* genus comprises around 50 plant species of the Agavaceae family from the American continent, but mainly from the United States, Mexico and Central America [1,2]. The species that belong to the Yucca genus have been used for years in traditional medicinal practices all over the world, but especially by Native Americans [1]. Subsequently, the research on this genus has made clear that they are plants with a high content of bioactive steroidal saponins [3].

**Figure 1:**
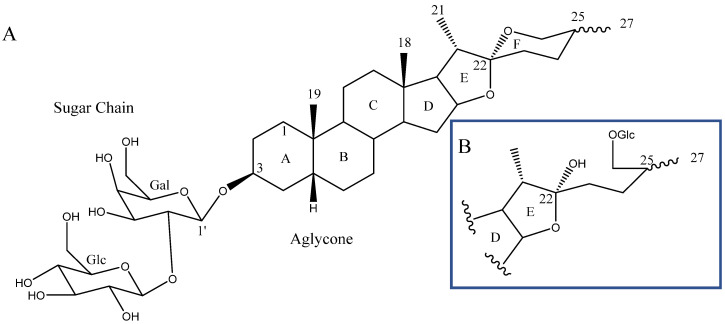
(**A**) Structure of a spirostanic saponin. (**B**) C-22 to C-27 carbons of a furostanic saponin.

Steroidal saponins are secondary metabolites of plant origin, with a high molecular weight and a complex two-part structure. One of these parts, known as the sapogenin or aglycone, is hydrophobic and consists of 27 carbon atoms arranged in five or six rings (named A–F). Depending on the nature of the aglycone attachment at C-22, they can be classified as either spirostane or furostane (Figure 1). The other moiety, the hydrophilic one, is usually formed by sugar residues with different monosaccharides linked by *O*-glycosidic bonds (Figure 1) [4,5]. In addition, two cholestane-type saponins have been uniquely described in the *Yucca* genus [6].

At present, some species of the *Yucca* genus and their saponins have been classified as Generally Recognized As Safe (GRAS) by the Food and Drug Administration of the United States (FDA) [1,7,8,9]. Therefore, several extracts and products derived from these plants have been commercialized as food supplements, parapharmaceutical complements, moisturizing agents, soil conditioners, etc. There are several producers of *Y. schidigera* products in the world such as Naturex, BAJA Yucca and American Extracts [10]. The saponins present in these plants have also been tested in separate studies, showing properties such as cytotoxic, phytotoxic, antifungal, molluscicidal, anti-inflammatory [11,12,13,14,15].

There are several reviews on the steroidal saponins from the Agavaceae family, including the *Yucca* genus, however, no work has been published to address this genus exclusively. The data that are usually collected in these studies are related to the structure and bioactivities of the saponins. An example of such work is that by Simmons-Boyce in 2007, where a total of 44 saponins found in the *Yucca* genus and their bioactivities were described [3].

Other papers have reviewed saponins from a specific species, such as *Y. schidigera* [9,16]. This species is the most widely marketed and the one with the largest number of studies. Even some very active saponins such as the so-called timosaponin AIII, present in *Y. macrocarpa* and *Y. gloriosa* and their bioactivities have been the subject of a number of reviews [17].

## 2. Structure of *Yucca* Saponins

### 2.1. Aglycone

**Figure 2:**
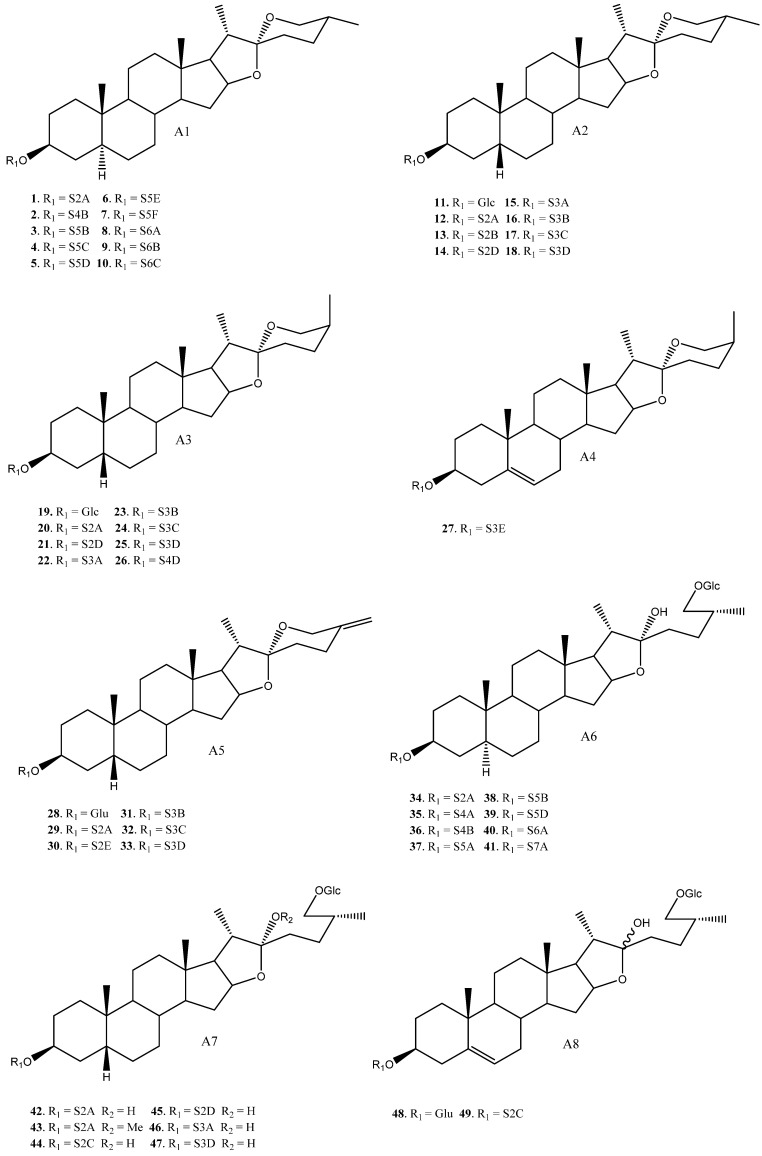

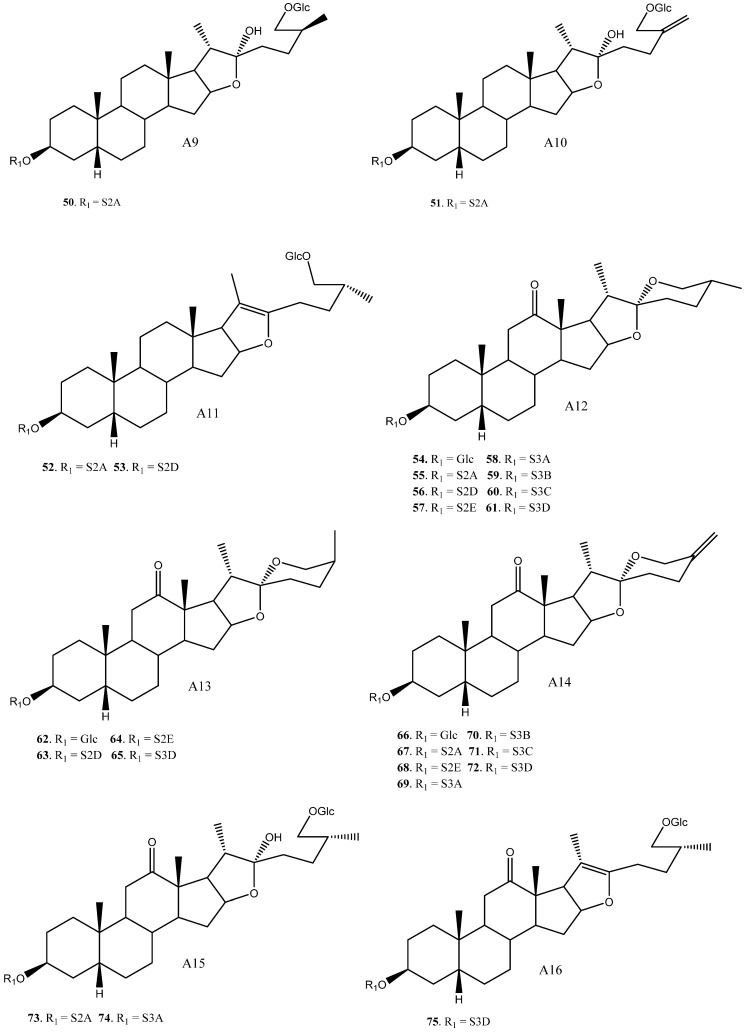

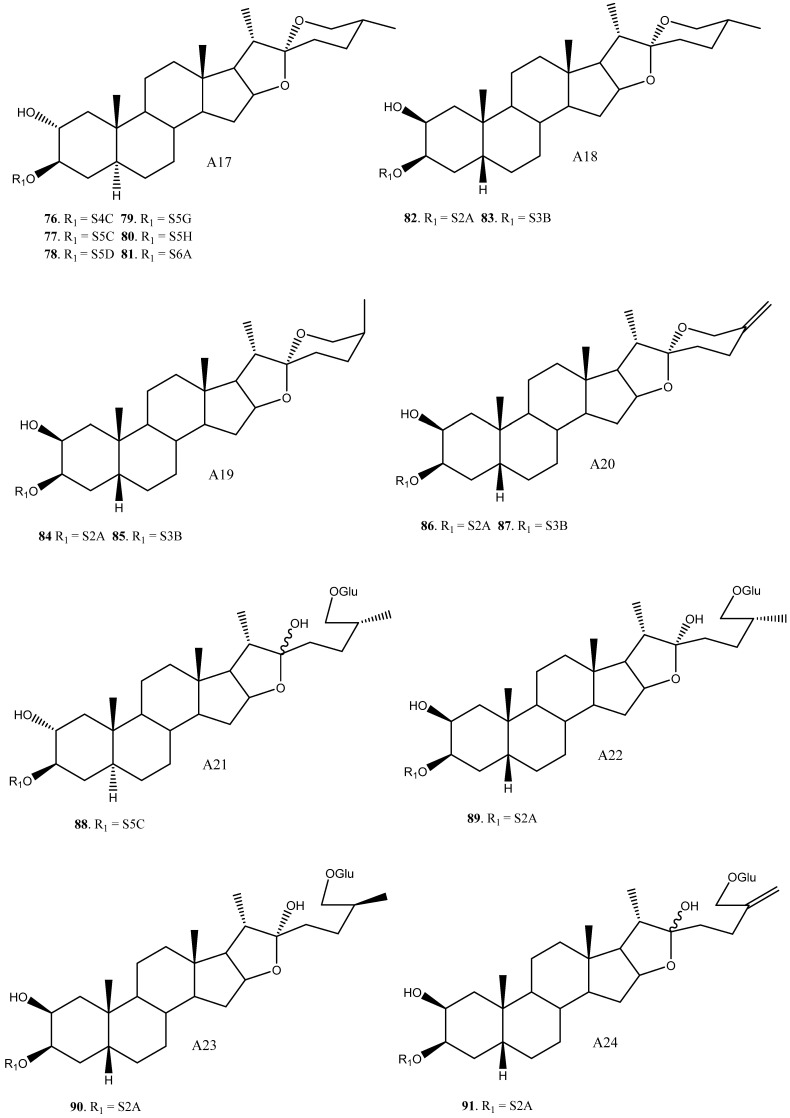

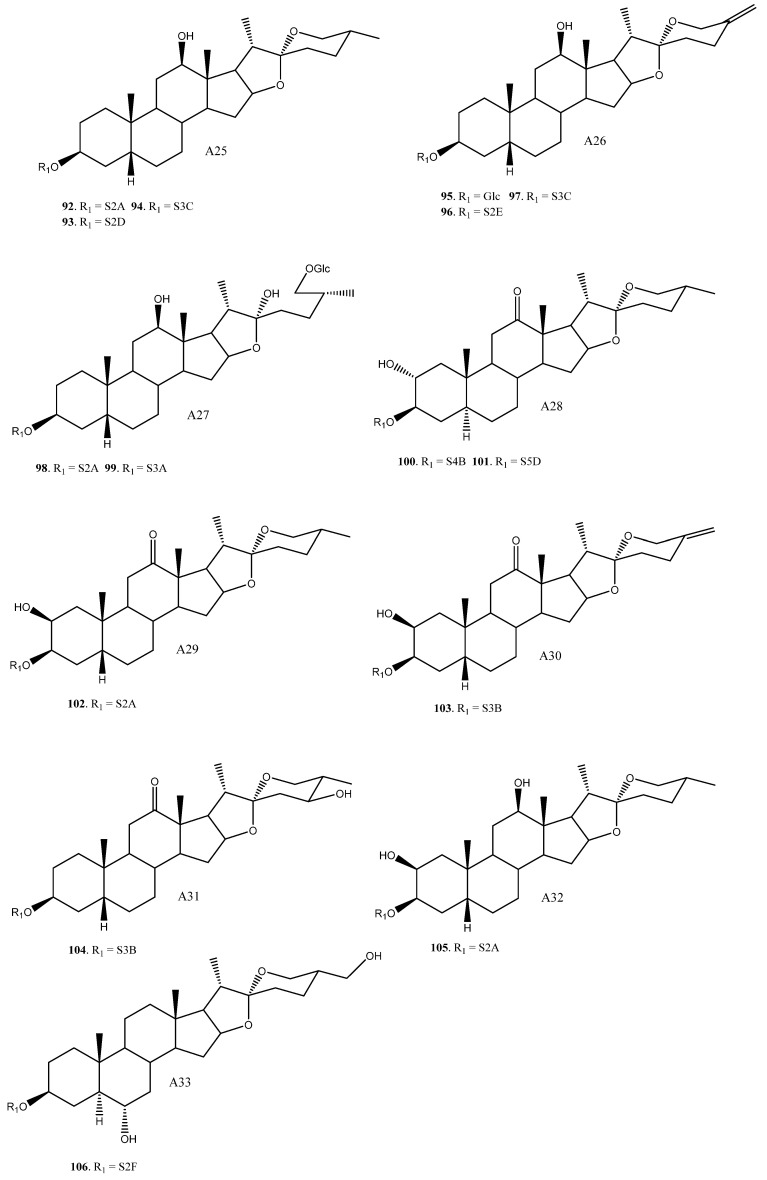
Structure of the spirostanic and furostanic saponins described in *Yucca* (**1**–**106**). The codes used for the aglycones (**A1**–**A33**) and the sugar chains (S#A-S#H) are included. Structures of sugars chains can be found further on Figures 6–8.

To date, 33 aglycones have been described as part of the saponins found in the *Yucca* genus [6,7,11,12,14,16,18,19,20,21,22,23,24,25,26,27,28,29,30,31,32,33,34,35,36,37], including spirostanic and furostanic saponins (Figure 2).

**Figure 3:**
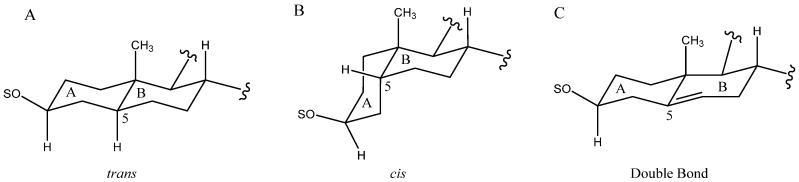
Structure of rings A and B with *trans* fusion (**A**), *cis* fusion (**B**) or double bonds between C-5 and C-6 (**C**).

The B to D rings are always fused with a *trans* arrangement, however, the A and B rings can also appear with a *cis* fusion as well as a double bond between the C-5 and C-6 carbons (Figure 3). Most common in *Yucca* is the *cis* fusion, which implies a β-arrangement of the H-5. However, depending on the species, one arrangement may be predominant over the other. For example, in *Y. desmetiana* the most abundant aglycone has a *trans* fusion of the A and B rings with H-5α [12,34], while in other species such as *Y. glauca* [11] or *Y. schidigera* [14,16,20] the H-5β form is the most abundant.

**Figure 4:**
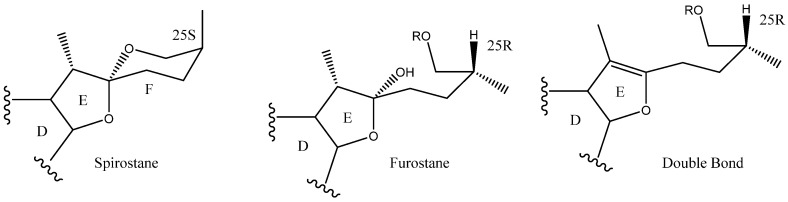
Structure of steroidal aglycones on the C-22.

The C-22 configuration determines the spirostanic or furostanic nature of a saponin. In the former, it is a spirostanic carbon, through which the E and F rings are linked, while in the furostanic ones C-22 is part of a hemiketal or enolic carbon with an open chain [6,16] (Figure 4). The most abundant aglycones in *Yucca* are the spirostanic ones, but this varies depending on the species. Thus, in the case of *Y. elephantipes* [19,36] or *Y. desmetiana* [12,34] furostanic aglycones are mostly isolated. It should be mentioned that C-22 is a chiral centre that always presents an *R* configuration in the case of the spirostanic derivatives, and it is also *R* in the case of the furostanic derivatives where the configuration has been described.

**Figure 5:**
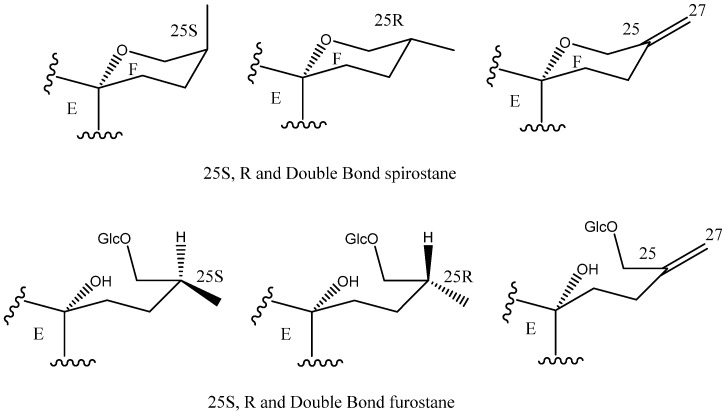
Structure of steroidal aglycones on the C-25.

C-25 is a chiral carbon which can have either *R* or *S* configuration, and a significant number of aglycones contain a double bond between C-25 and C-27 (Figure 5).

Regarding the oxygenation in *Yucca* aglycones, we find them at carbons 2, 12, 24 and 27. On position 2, hydroxyl groups appear in α and β arrangements, and their configuration always coincides with that of H-5, which allows us to suggest that this structural outcome results from their biosynthesis pathway. On position 12, a carbonyl group and, in some cases, a hydroxyl group with a β-arrangement are most frequently observed. Moreover, only one spirostanic saponin (**104**), described in *Y. glauca* [11], is hydroxylated at C-24 and another found in *Y. smalliana* (**106**) is hydroxylated at C-27 [33]. It is interesting to note that all of the hydroxyl groups that substitute the *Yucca* aglycones present an equatorial arrangement within the rings that contain them.

### 2.2. Sugar Chains

Saponins can be classified according to the number of sugar chains that they contain. Those which have only one chain are called monodesmosidic, and with respect to the saponins in the *Yucca* genus, this is located on position C-3. The saponins with two sugar chains are called bidesmosidic, and in the genus *Yucca* coincide with the furostanic saponins, which have a glucose unit on C-26 in addition to the sugar chain on C-3 [5].

In addition to glucose, 22 different sugar chains have been described in *Yucca*. These are linked to aglycones on the C-3 position [6,7,11,12,14,16,18,19,20,21,22,23,24,25,26,27,28,29,30,31,32,33,34,35,36,37]. The chains described in this genus possess up to seven monosaccharides.

**Figure 6:**
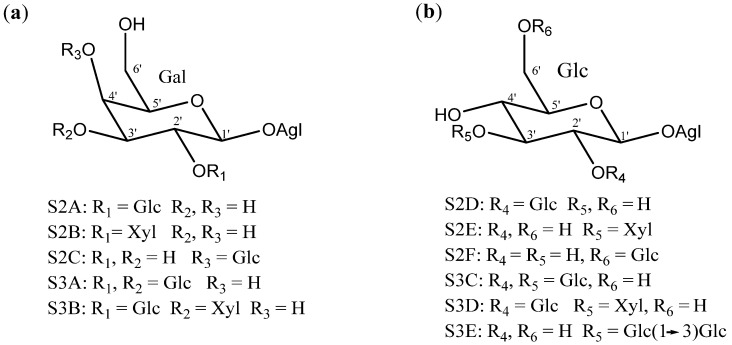
Structure of the disaccharides (S2A to S2F) and trisaccharides (S3A to S3E) present in *Yucca* (**a**) when β-d-galactopyranose is linked at C-3 of the aglycone and (**b**) when is a β-d-glucopyranose. Gal = galactose; Glc = glucose and Xyl = xylose.

Disaccharides and trisaccharides exhibit a wide structural diversity (Figure 6). The monomer that bonds directly to the C-3 of the aglycone can be either β-d-galactopyranose or β-d-glucopyranose. These monosaccharides are linked by glycosidic bonding to β-d-glucopyranoses or β-d-xylopyranoses on the positions 2’, 3’ or 4’, in the case of galactose, and 2’ or 3’, in the case of glucose. The most common disaccharide in *Yucca* is glucopyranosyloxy (1→2) galactopyranoside (S2A).

**Figure 7:**
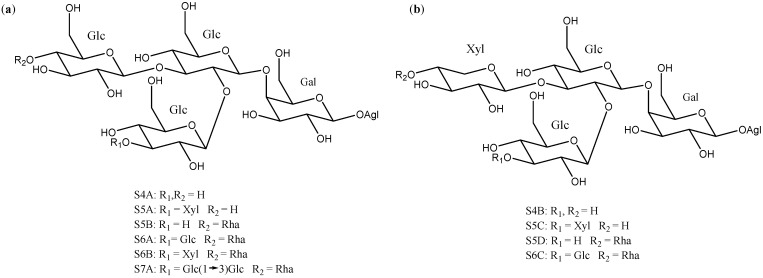
Structure of *Yucca* polysaccharides S4A to S7A (**a**) with a β-d-galactopyranose and three β-d-glucopyranoses and (**b**) with a β-d-galactopyranose, two β-d-glucopyranoses and a β-d-xylopyranose core. Gal = galactose; Glc = glucose; Xyl = xylose and Rha = rhamnose.

Sugar chains of four or more units have also been described in *Yucca*, although they are less abundant than the above described (Figure 7). Ten of them have the same core, which consists of a β-d-galactopyranose and two β-d-glucopyranoses, to which β-d-glucopyranoses, β-d-xylopyranoses or α-l-ramnopyranoses are linked at different positions to form the actual polysaccharide.

**Figure 8:**
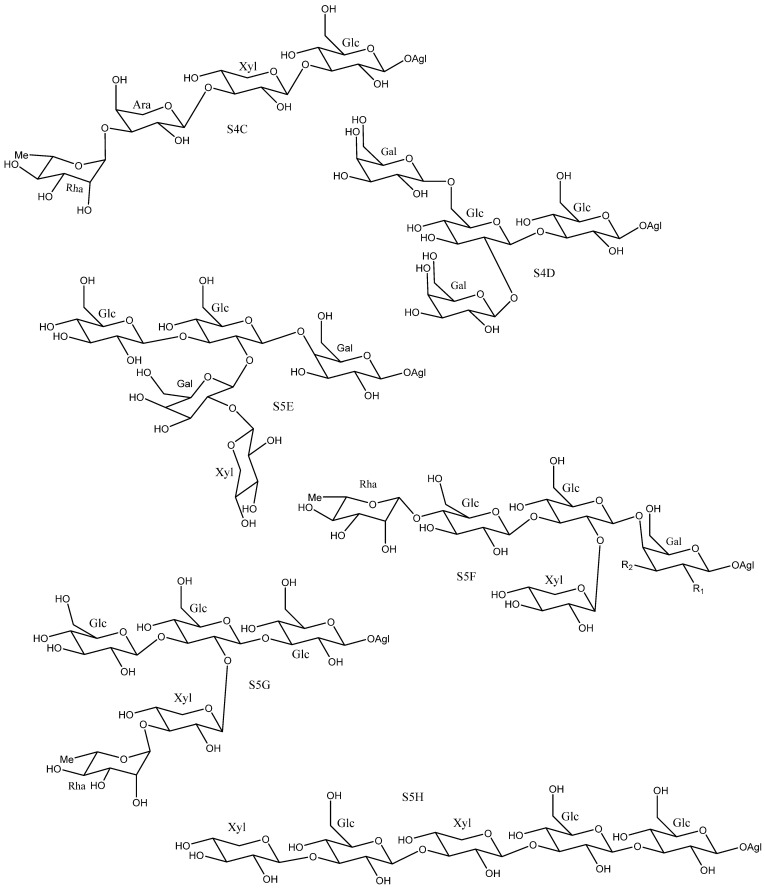
*Yucca* polysaccharides with unusual structures.

On the other hand, other polysaccharides, such as S4C [25], S4D [18], S5G [24] and S5H [26], which present structures that do not follow this pattern have also been described (Figure 8), however their characterization was carried out, in some cases, using the non- fully developed structural elucidation techniques that were available for saponins before the early 1990s. Hence, the sugar chains S4C, S5G and S5H were reported in different publications on the saponins present in *Y. aloifolia* [24,25,26]. The structures of these saponins are very different from each other, which leads us to suspect errors in their identification. In fact, subsequent investigations that used mono- and bidimensional NMR techniques showed that, the larger sugar chains were derived from the simpler ones in saponins isolated from the same species. Thus, the larger sugar chains are formed by addition of monosaccharides to the simpler core [11,16,29]. This reinforces the idea that previous determinations of these structures are rather questionable.

### 2.3. Saponins in the Yucca Genus

106 spirostanic and furostanic saponins (Table 1) have been reported in the species of the *Yucca* genus that have been studied, namely *Y. gloriosa*, *Y. glauca*, *Y. schidigera*, *Y. aloifolia*, *Y. desmetiana*, *Y. elephantipes Y. macrocarpa*, and *Y. smalliana* [6,7,11,12,14,16,18,19,20,21,22,23,24,25,26,27,28,29,30,31,32,33,34,35,36,37]. Only a few of these have been studied in depth, which lead us to conclude that the saponins from the *Yucca* genus remain a rather underexplored field.

Overall, based on the data available, it is observed that some saponins such as **12**, **16**, **42**, **55** and **82** are described in three or four different species, however, the general trend is that most of the saponins are found in only one or two species.

The saponins with a H-5β aglycone arrangement have a sugar chain of three or fewer units attached to C-3. For example, in *Y. schidigera* [14,16,20], the majority of the saponins are 5β-spirostanic and all of them have chains formed by two or three monosaccharides. In other species, such as *Y. aloifolia* [24,25,26], the isolated saponins are 5α-spirostanic with sugar chains of four and five units. In the case of *Y. gloriosa*, a wide variety of spirostanic and furostanic saponins with sugar chains ranging from two to seven units have been isolated. In particular, it is in this species that the largest sugar chains have been described [29]. The same trend is also true for *Y. gloriosa*, where the saponins with a β-arrangement at H-5 have chains formed by less than three polysaccharides, while those with an α-arrangement at H-5 present the longest chains.

This trend has also been observed in the saponins from the *Agave* genus [91] and there seems to be a correlation between the fusion of the A and B rings and the size of the sugar chain in the saponins, which seems to be related to some spatial effect that occurs between the *cis* fusion aglycones and the more voluminous chains.

**Figure 9:**
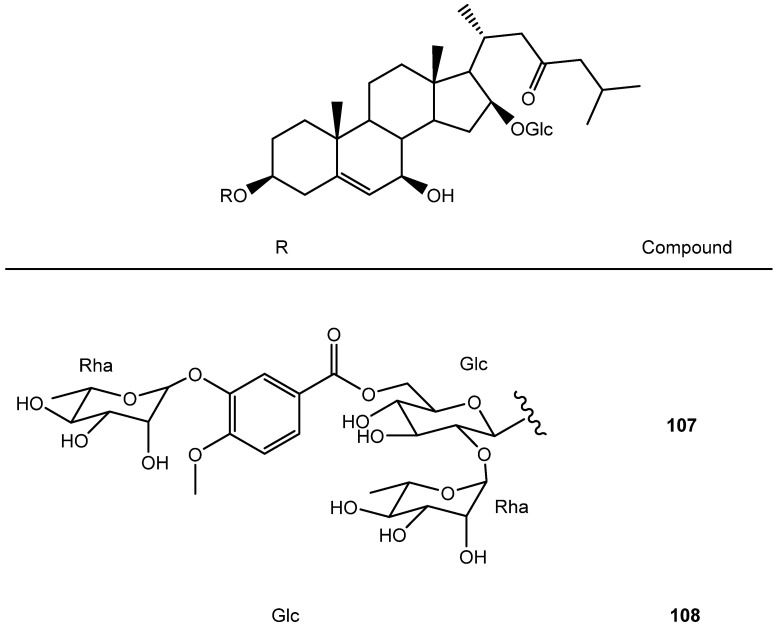
Structure of cholestane-type saponins from *Yucca*.

Two cholestane-type saponins (**107**–**108**) have also been described in the *Yucca* genus [6] (Figure 9).

## 3. Bioactivities by Yucca Saponins

In addition to compiling the activities shown by extracts from species of the genus *Yucca*, a bibliographic search has been carried out on the bioactivity of the 108 saponins described in the genus. The most significant results are shown in Table 1. Only 69 of these saponins have been tested and their biological activities that have been most studied are cytotoxic, antifungal, anti-inflammatory and effects on platelet aggregation.

Some extracts such as those from *Y. gloriosa* flowers or dried leaves [31,32], as well as *Y. schidigera* stems [14] showed antifungal activity against a large number of yeasts and fungi, which makes of these extracts suitable candidates to be used as food additives or preservatives [92]. In fact, *Y. schidigera* extract is commercially available and has been classified as GRAS by the FDA (USA) [9] and it appears on the “List of Existing Food Additives” in Japan, so that it can be used there as a human dietary additive [92].

In addition to this, certain saponins and extracts from *Y. schidigera* have been proposed to improve the health conditions of aquatic organisms [93,94] as well as the immune response of broilers [95]. Moreover, when *Y. schidigera* extract is administered to ruminants, or horses, there is a reduction in the growth of protozoa and Gram-positive bacteria which are associated in some cases to colitis, and other processes leading to inflammatory diseases [96]. In addition, some studies have associated the regular ingestion of *Yucca* extracts with a reduction in cholesterol levels in hypercholesterolemic humans [97].

The saponins isolated from different extracts of the genus *Yucca* have also been studied. Interesting cytotoxic activities against various cancer cell lines have been described for saponins from *Y. glauca* [11], *Y. desmetiana* [34] and *Y. schidigera* [28]. Antifungal activity has also been tested on pure saponins and epimers mixtures on the C-25 position in *Y. gloriosa* [31], *Y. elephantipes* [36] and *Y. schidigera* [14], showing very promising activity values. These two activities have been the most frequently tested on the saponins from *Yucca*, including studies on structure-activity relationships (SAR). Apart from these activities, two saponins from *Y. desmetiana* [12] and one from *Y. aloifolia* [25] presented molluscidal activity.

Some of the saponins described in the genus *Yucca* have been tested for bioactivities such as anti-inflammatory after being isolated from other species. Inflammation [98], a defensive physiological response to noxious stimuli, is a highly complex process involving multiple cells, signaling pathways and molecules. Although inflammation is a natural and beneficial process, an excessive inflammatory response may lead to chronic damage and adverse reactions. Some saponins have been tested against certain processes involved in the inflammatory response, such as the release of nitric oxide (NO) [72,86], production of inflammatory interleukins [72,86], recruitment of immune system cells [59] or inhibition of specific metabolic pathways [15,78,86] and in some cases, they exhibited rather promising results. Some of the chronic conditions that may result from inflammatory processes are osteoporosis [64] or cardiovascular damages due to high blood glucose levels [99]. Additionally, neurodegenerative diseases such as Alzheimer’s or Parkinson’s diseases are among other possible undesirable conditions [63,75,90]. Three of the saponins present in *Yucca*, namely timosaponin AIII (**20**), timosaponin BII (**50**), and degalactotigonin (**2**), have been tested more extensively and have been distinguished by their intense and varied activity.

### 3.1. Cytotoxicity: Study of the Structure-Activity Relationships (SAR)

Cytotoxicity is one of the activities that has been most frequently tested in natural products and is often described in *Yucca* saponins. In most cases, these molecules are evaluated for their ability to be toxic by producing visible damage to various cancer cell lines [100]. Such damage may occur through various pathways or mechanisms of action, being rather frequent the regulation of cellular apoptotic pathways [11,71].

In some cytotoxicity studies, inhibitory concentration (IC_50_) values are used as a benchmark to assess the cytotoxic effect of the active substance. IC_50_ is the concentration level of a compound that is required to inhibit growth by 50% in comparison to untreated cells [11,34].

Yokosuka et al. [11] tested 20 spirostanic and furostanic saponins extracted from *Y. glauca* (Table 2 and Table 3) against human acute myelogenous leukemia (HL-60) and human lung adenocarcinome (A549) cell lines. A connection between the structure of these 20 saponins and their activity could be identified [11].

As a bioguided isolation method, all the saponins were first tested against HL-60 cells and only the active ones were tested against the A549 line. Cisplatin and Etoposide were used as the control drugs. All the tested saponins from *Y. glauca* present a *cis* fusion of the A and B rings (H-5β) with residues of three or less sugar units attached to C-3. Moreover, they differ regarding the oxygenation of the aglycone, by the nature of the C-25, their furostanic or spirostanic structure, and in the sugar chains.

The saponins that presented IC_50_ values lower than 20 µM against both cell lines (**12**, **16**, **29**, **31**, **42**, **50** and **51**), had in common the absence of oxygenation at the aglycone, while those with a hydroxyl group on C-2 (**82**, **86**, **89**, **90** and **91**), were only active against HL-60 [11]. Finally, oxygenation on the C-12 position, did not favor cytotoxicity, since all the saponins of this type presented IC_50_ values above 20 µM against the HL-60 cell line.

If the influence attributable to their spirostanic or furostanic nature is examined, the comparison of some pairs of compounds such as **29** vs. **51**, **12** vs. **42, 86** vs. **91** and **82** vs. **89** indicate that the spirostanic derivatives were more active in all the cases [11].

The influence of the *R* or *S* configuration of C-25 and even the presence of a double bond between this carbon and C-27, does not appear to be significant, since only slight variations of the IC_50_ values and no definite trend were observed.

Regarding the type of sugar chain of the tested saponins, the vast majority of active saponins presented a glucopyranosyl (1–2) galactopyranoside (S2A) chain, or a derivative of it such as the S3B chain, which presents a xylopyranosyl unit on the C-3 of the galactose (31). On the other hand, β-d-glucopyranosyloxy (1→4) β-d-galactopyranoside (S2C) chains give rise to a less active saponin (**42** vs. **44**), especially against the A549 cell line.

The most active saponin **29** and its furostane derivative **51** were assayed to determine their ability to induce apoptosis of the HL-60 cells. The assay was based on measuring the activity of caspase-3 [11]. Thus, it could be determined that spirostanic saponin **29** was effective to significantly produce apoptosis in a shorter period of time (6 h) than saponin **51** (16 h) and with higher caspase-3 activity values, which were close to those presented by the control substance (etoposide) [11].

In a different study by Qu et al. [28], the cytotoxic activity of 12 saponins isolated from *Y. schidigera* (Table 4) was analyzed against human colon adenocarcinoma cell line (SW620).

It can be observed that the saponins with carbonyl substituents on C-12 (**54**, **56**, **57**, **62**, **63** and **64**) are generally less active than those without such functionalization (**11**, **14**, **18**, **19**, **21** and **25**), which is in agreement with the reports from previous studies. On the other hand, it is noteworthy that in the saponins with a non-functionalized C-12, the *S* epimers on C-25 are more active. The difference in activity between *R* and *S* epimers, which in some cases is rather noticeable, had not been observed in previous studies. The size of the sugar chain has an influence on the activity displayed, being compounds with three-unit chains the most active ones (**25**, 12.02 µM) [28]. 

Sun et al. [65], demonstrated with saponins from *Anemarrhena asphodeloides*, which included some of the saponins contained in *Yucca*, that glycosylation is considered essential regarding cytotoxic activity, since when the aglycones were tested separately, they did not exhibit any activity.

### 3.2. Antifungal: Study of the Structure-Activity Relationships (SAR)

Antifungal or antimycotic activity is the effect that certain substances have on fungi, preventing their proliferation, development and, in some cases, causing their death. The activity is represented by IC_50_ values, or Minimum Inhibitory Concentration (MIC) values, i.e., the minimum amount of compound at which the organism does not appear to grow [14].

Miyakoshi et al. [14], tested a collection of saponins, which included six pure saponins and eight mixtures of C-25 epimers, all of them extracted from *Y. schidigera* (Table 5 and Table 6) against a number of selected organisms: *Saccharomyces cerevisiae* IFO 203 (Sc), *Candida albicans* TIMM 0134 (Ca), *Hansenula anomala* HUT 7083 (Ha), *Pichia nakazawae* HUT 1688 (Pn), *Kloeckera apiculata* IFO 154 (Ka) and *Debaryomyces hansenii* IFO18 (Dh).

The isolation process of the saponins was conducted following a bioguided procedure, on which as the fraction containing furostanic saponins did not show activity, therefore, the study was focused on the spirostanic saponins [14]. On the other hand, the spirostanic saponins were hydrolyzed and the aglycones were tested and found to be inactive. It has been determined that the participation of both parts of the saponin, the sugar chain and the aglycone, is required to present any relevant activity.

The results from the tests with spirostanic saponins and their mixtures showed that the presence of oxidations on the C-2 and C-12 positions (hydroxyl and carbonyl groups) resulted in a drastic drop or even the absence of activity. On the other hand, those without such oxidations present better MIC values [14].

If the saponins are compared, in the ones that only differ in the presence or absence of a double bond between C-25 and C-27, no general trends can be observed for all the tested organisms, except for *Kloeckera apiculata* IFO 154 (Ka) and *Candida albicans* TIMM 0134 (Ca) where the absence of such a double bond results in a sharp decrease in activity (cf. **33** vs. **18** + **25**).

With regard to the nature of the sugar chain, four types of trisaccharides and one disaccharide have been tested for saponins without oxygenation in the aglycone. In the case of saponins **31, 32** and **33**, with a double bond between carbons C-25 and C-27 and whose only difference is the nature of the trisaccharide, we observed that saponin **31** is the most active, with particular relevance for *Kloeckera apiculata* IFO 154 (Ka) and *Debaryomyces hansenii* IFO18 (Dh). However, their saponin analogues without a double bond, do not present such a marked difference, with the **18** + **25** saponin mixture showing the highest activity, especially on *Candida albicans* TIMM 0134 (Ca). The sugar chain of saponins **33, 18** and **25** is a β-d-xylpyranosyl(1→3)[β-d-glucopyranosyl(1→2) β-d-glucopyranoside] (S3D) which differs from the S3B chain (**31, 16, 23**) where the innermost monosaccharide is a β-d-galactopyranose. On the other hand, the S3C chain (**32, 17, 24**) has a β-d-glucopyranose unit instead of the β-d-xylopyranose that is found in S3B. Finally, the **22** + **15** saponin mixture with the S3A chain, which is similar to S3C although with a β-d-galactopyranose as the innermost monosaccharide, exhibited activity values against all of the organisms tested. Although it could be concluded that the nature of the sugar chain affects the activity values reached by the tested spirostanic saponins, no clear trends were observed in relation to the nature of the constituent monosaccharides.

### 3.3. Selection of Active Saponins

#### 3.3.1. Degalactotigonin (**2**)

**Figure 10:**
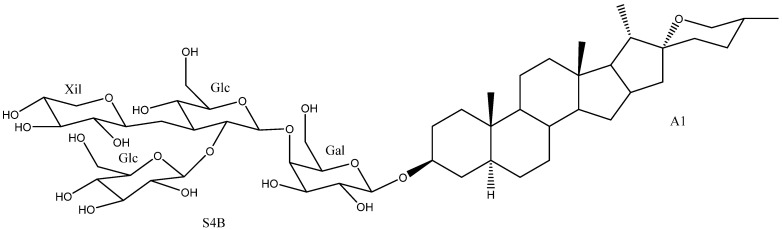
Degalactotigonin (**2**).

Degalactotigonin (**2**, Figure 10) has been isolated from *Y. gloriosa* flowers [6,7], but also from other species or genera [39,40,42,43,44,45,46,47,48].

Degalactotigonin (**2**) from different species has been tested for cytotoxic, antifungal, spermicidal and platelet aggregation effects. Regarding its antifungal activity, it has been tested against several fungal strains, reaching an IC_50_ value of 0.40 µM for *Candida neoformans*, which exceeds the expected performance by the control fungicide amphotericin B (0.50 µM) [39]. In addition, it was found to be effective for the control of black spot disease caused by *Alternaria brassicicola* that affects cabbage leaves, as it inhibits spore germination [40].

Other studies [39,44,45,46,47,48], have evaluated its cytotoxic capacity. It has been tested against 26 cell lines, being active in 21 of them with IC_50_ values ranging from 0.03 to 31.46 µM. The best results were obtained against human lung cancer (PC-12), and human colon cancer (HCT-116) cell lines with IC_50_ values of 0.03 and 0.22 µM respectively. These values were 10 times higher than those reported for the control substance, cisplatin, with 0.33 and 2.25 µM, respectively [46].

Its spermicidal capacity has been confirmed, presenting an EC_50_ value of 29.8 µM and being much more effective, selective and safe than the control substance, nonoxynol-9 (78.34 µM) [43]. In addition, the mixture of saponin **2** with gitonin, which differs from the first one in a hydroxyl group on the C-2 position of the aglycone, has shown a moderate capacity as a platelet aggregator, with 74% maximum induction percentage at a concentration of 250 µM [42]. Finally, a pharmaceutical preparation to prevent and relieve flu symptoms containing degalactotigonin (**2**) has been patented [41].

#### 3.3.2. Timosaponin AIII (**20**)

**Figure 11:**
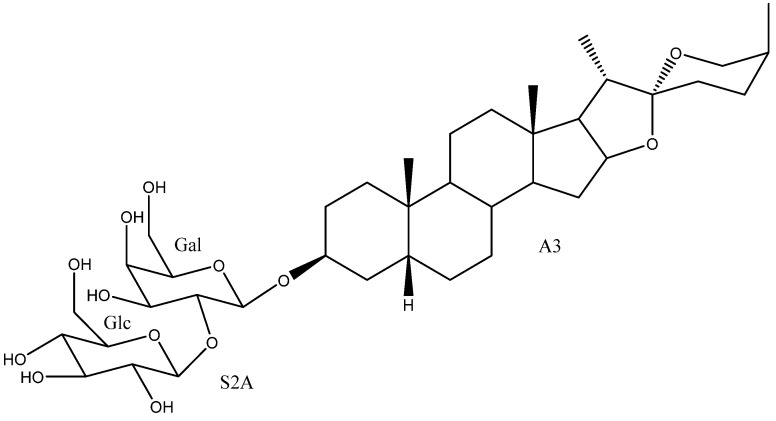
Timosaponin AIII (**20**).

Timosaponin AIII (**20**, Figure 11) has been identified in *Y. gloriosa* [6] and in *Y. macrocarpa* [37], as well as in other genera, and a considerable number of studies on this saponin have described five bioactivities (Table 7). 

Although timosaponin AIII (**20**) was not included in previous studies on the structure-activity relationship (SAR), this saponin and its epimer on C-25 (**12**) were synthesized [38] and tested on human epithelial cervical cancer cell (HeLa) and showed similar activity values. Timosaponin AIII (**20**) is notable for its exceptional cytotoxic and anti-inflammatory properties. In particular, it showed cytotoxic activity against the Hep3B liver cancer cell line (0.35 µM), where its IC_50_ was almost 100 times lower than that of the control substance 5-fluorouracil (22.99 µM) [65]. On the other hand, its anti-inflammatory capacity is quite potent too, being able to reduce the total number of inflammatory cells in the broncho-alveolar lavage fluid (BALF) by up to 64%, which is similar to that of the control compound, dexomethasone [59].

Timosaponin AIII (**20**) is included in a patent where it is proposed for cosmetic use as an active ingredient in a moisturizing cream [101] and in another patent where it is proposed to be included together with timosaponins BI and AI in a drug against cognitive disorders [102].

#### 3.3.3. Timosaponin BII (**50**)

**Figure 12:**
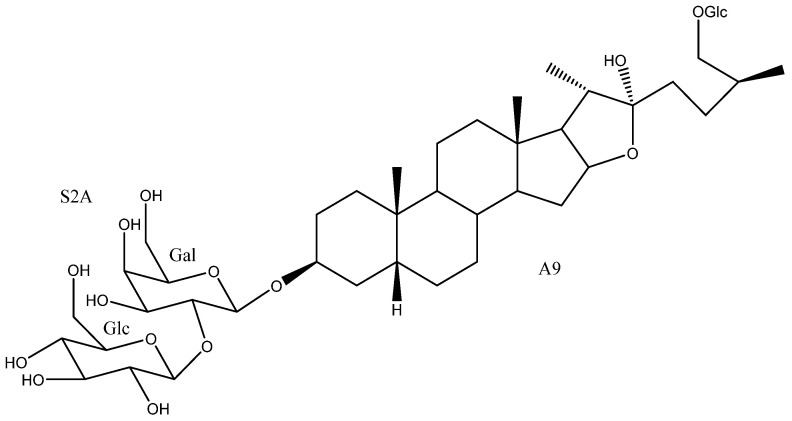
Timosaponin BII (**50**).

Timosaponin BII (**50**) (Figure 12) has been isolated from *Y. glauca* [11] and, it is the furostanic derivative of timosaponin AIII (**20**). Seven activities have been tested and are shown in Table 8.

Among the activities tested for this saponin, the most notable results were obtained with regard to its cytotoxic and anti-inflammatory capacities. Timosaponin BII (**50**) was tested against the HL-60 and A549 cell lines together with other furostanic saponins, showing IC_50_ values of 3.3 and 9.3 µM, respectively. These values are among the best in the study and are comparable to those of the control drug, cisplatin (1.7 and 2.1 µM) [11]. On the other hand, it has been demonstrated that timosaponin BII (**50**)-loaded nanofibers are capable of inhibiting human hepatocellular carcinoma SMMC-7721 cells, both in vivo and in vitro. More specifically, 10–15% volume weight percentages of timosaponin BII nanofibers can generate a potent growth inhibition against SMMC-7721 cells [76].

Timosaponins AIII (**20**) and BII (**50**) [15] were tested for their ability to selectively inhibit two molecules closely related to inflammatory metabolic pathways (2-COX and 5-LOX). Timosaponin BII (**50**) showed a better IC_50_ value for 2-COX inhibition (0.77 vs. 1.82 µM), whereas timosaponin AIII (**20**) proved to be more effective to inhibit 5-LOX (1.21 vs. 1.57 µM).

Timosaponin BII (**50**) has been patented as one of the main active compounds in a vaginal antifungal cream [105]. Moreover, together with other timosaponins including AIII (**20**) and N (**90**) it has been proposed as a preparation for the treatment of viral myocarditis [62].

## 4. Analytical Methods for the Steroidal Saponins in *Yucca*

As it was previously mentioned, *Yucca* saponins are of particular interest to the cosmetic, pharmaceutical and beverage industries as well as for the animal feed industry, being a highlighted feature their foaming activity. Consequently, *Yucca* extracts have been the focus of attention for numerous researchers. One of the main sources of saponins for commercial purposes and also one of the best known species is *Y. schidigera* [9,106]. Saponins present a rather complex structure, generally appearing as a mixture of isomers whose separation and quantification still represent a considerable challenge. Environmental conditions and plant development stage result in varying metabolite content [107]. These variations represent an additional difficulty when trying to determine quantity and quality of these compounds for commercial use. Conventional analytical methods used for this purpose include foam height measurements, spectrophotometric and gravimetric methods. Foam properties (a highly significant feature required in some chemical, foods, cosmetic and pharmaceutical processes) can be directly related to its saponin content [21]. These compounds are capable of reducing surface tension and improve the foamability of some aqueous solutions, even when added at low concentrations [108,109]. However, classifying saponins into ionic or non-ionic surfactants as the basis to determine their foaming properties does not seem to be the appropriate procedure, since these compounds are not necessarily sensitive to changes in ionic strength [110]. Besides, the volume and quality of foam produced by any method depends on several and complex factors [111]. Moreover, gravimetric methods base their measurement on butanol fractions, which presents a double-edged difficulty. On the one hand, butanol has no affinity with polar saponins and, on the other, this solvent may extract other kinds of compounds such as polyphenols or organic acids. This would result in an inaccurate determination of the saponin content [8,106].

Spectrophotometric methods, such as UV, have also failed to prove accurate, since saponins do not have a strong UV chromophore that can be detected by UV analysis [6]. In order to improve results, saponins have been derivatized to form colored substances. Thus, using this method, a spectrophotometric assay claimed to have determined the deglycosylation of steroidal saponins into sapogenin within the ruminal fluid of cattle that had been fed with *Y. schidigera* saponins. This assay. which was a modified version of the method described by Baccou et al. [112] showed that rumen bacteria are capable of degrading saponin into sapogenin or aglycone but cannot degrade the aglycone core. This colorimetric method is based on the reactions that take place with anisaldehyde, sulphuric acid and ethyl acetate to form chromophores [113].

Other chromatographic methods have been widely used for the analysis of saponins. Thus, a low-cost method that provides a preliminary assessment and verification of the saponins found in some commercial products containing *Y. schidigera* has been recently developed. This method uses TLC silica gel 60 F_254_, CHCl_3_:CH_3_OH:H_2_O (35:14:1) and 10% sulphuric acid-ethanol solution to improve plate visualization. The saponins became apparent under these chromatographic conditions when the retention factor was 0.38 or lower [114]. In another study, despite the difficulty to separate isomers, six pairs of 25 (*R*/*S*)-spirostanol saponin diastereomers from *Y. schidigera* were successfully separated through HPLC using a C_30_ column and their structure was unequivocally confirmed by NMR analysis [28]. On the other hand, 25 (*R*/*S*)-spirostanol saponin diastereomers have been successfully separated using supercritical fluid chromatography [115].

Mass spectrometry represents an effective detection method where selectivity and specificity can be improved by tandem mass spectrometry. Thus, in the last few years, different methods using tandem mass spectrometry have been developed for a quick and reliable determination of saponins [8]. It should also be mentioned that the use of mass spectrometry as a single technique for the determination of saponins presents some drawbacks, since it does not allow to differentiate isomers, and neither the position of the sugar chain link on the aglycone, nor the connectivity between sugar residues can be determined. Mass spectrometry and chromatographic methods are not often used as a single technique for the determination of these compounds, but rather in combination or coupled to other techniques. In this way, the detection and quantification of the nineteen steroidal saponins contained in a commercial syrup and in a bark’s extract from *Y. schidigera* have been carried out by LC-MS. Thus, twelve saponins were confirmed by comparing their fragmentation patterns against their corresponding standards and their spectrometric data, while the rest of the saponins were structurally proposed [8]. Furthermore, Montoro et al. developed a method for the quantitative analysis of the steroidal saponins in Y. gloriosa flowers real samples using HPLC coupled to tandem mass spectrometry [7]. LC-ESI-MS analysis has also been applied to the detection of the saponins contained in different *Yucca aloifolia* varieties imported into Egypt [116]. HPLC/ELSD is another accurate analysis and quantification method that has been applied to determine the total steroidal saponin content in *Y. schidigera* extracts. This is a fast and reliable technique that allows the quantification of molecules that do not contain a chromophore group, like in the case of saponins [106]. In the same way, Sastre et al. described a method for the analysis of the four major saponins in certain commercial samples of *Y. schidigera* by means of HPLC coupled to an evaporative light scattering detector (ELSD) and matrix-assisted laser desorption/ionization time-of-flight (MALDI-TOF) mass spectrometry [117]. Recently, Ruan and co-workers have identified 110 spirostanol saponins by multi-phase liquid chromatography (MPLC) combined with MS/MS. This approach improves chromatographic peak capacity and became the first method to provide a comprehensive characterization of spirostanol saponins from *Y. schidigera* [118].

NMR-based metabolomics is another reliable tool, which in combination with certain conventional quality control methods, can perform an effective initial screening of the raw materials in *Yucca* extracts [107]. For instance, a general approach to the identification and structural elucidation of the steroidal saponins in *Y. filamentosa* has been performed successfully. No full signal assignments were required to identify the compounds, since MS analyses were carried out firstly and then their results were supplemented with NMR data [27].

*Yucca* has proven to be a valuable source of steroidal saponins and its commercial extracts have been classified as GRAS by the US FDA. It is, therefore, widely used as a surfactant additive for the manufacturing of beverages, animal feed, cosmetics and some agricultural products. Despite its increasing applications, a method to evaluate and control the quality of the saponins contained in this plant genus and often incorporated to commercial products is still an unresolved issue. These metabolites are usually found in complex mixtures, structurally related and their separation is still a task [119]. Thereby, the recent methods described for their identification are based in different techniques coupled between them, being the most used, LC-MS technique. Moreover, the most of the studies have been carried out with extracts of *Y. schidigera*, being this species one of the major commercial sources of saponins.

## 5. Materials and Methods

The bibliographic searches were carried out mainly using the SciFinder database. The first search used the keywords ”*Yucca*”, “saponin”, “steroidal”, “spirostanic glycoside”. A second search using the respective Chemical Abstracts Service (CAS) numbers was carried out for the bioactivities of the 108 saponins described in the genus *Yucca*. The filter “biological study” was used. In order to retrieve information on the analytical methods used for the analysis of saponins, a third bibliographic search was carried out using the keywords “analysis”, “analytical methods”, “saponins” and “*Yucca*”.

## 6. Conclusions

The plants from the *Yucca* genus are generally recognized as a valuable source of steroidal saponins. This explains why their commercial extracts have been classified by the United States Food and Drug Administration as GRAS. Consequently, they have been widely used as surfactant additives by different industries, such as beverage, animal feed, cosmetics or agriculture.

A total of 108 saponins have been described in eight species of this genus. The most common structural features of *Yucca* saponins are a spirostanic aglycone with *cis* fusion of the A and B rings, and sugar chains of three or less sugar residues. This type of structure is associated to potent cytotoxic and antifungal activity as long as the aglycone is free from oxygenated functional groups.

Despite the increasing number of saponin applications, an accurate and reliable method to assess and control their quality is still to be fully developed. This is probably due to the difficulties to carry out their separation, since they are usually found in complex mixtures that are structurally related [119].

If we are to describe the state of things at present, we should mention that *Y. schidigera* is the species most often used for commercial extracts and that LC-MS is the most widely used technique for the identification, quantification and quality control of saponins in *Yucca* or *Yucca* extracts. The identification of saponins in *Yucca* genus is still a relatively unexplored field and further investigations would allow to know new structures and potential bioactivities. Furthermore, there is the need to develop methods that allow a reliable and suitable analysis of the saponins for quality control of commercial *Yucca* samples.

## Figures and Tables

**Table 1:** Spirostanic and furostanic saponins from *Yucca*, their origin and bioactivities.

N°	Name	Aglycone	Sugar Chain	*Yucca* Species	Bioactivities	Refs.
**1**	Yuccaloeside A	A1	S2A	*Y. gloriosa*	Cytotoxic (HeLa IC_50_ = 12 μM)	[7,29,32,38]
**2**	Degalactotigonin	A1	S4B	*Y. gloriosa*	See Section 3.3.1	[7,29,39,40,41,42,43,44,45,46,47,48]
**3**	Yuccaloeside B	A1	S5B	*Y. gloriosa*	Cytotoxic (MSC-2 y HGF LD_50_ = 1.9 y 20 μM) Antifungal (Various strings, MIC 0.78→100 μM)	[7,31,32,49]
**4**		A1	S5C	*Y. gloriosa*	Cytotoxic (Vero IC_50_ = 7.5 μM y HeLa IC_50_ = 7.2 μM) Antifungal (Various strings IC_50_ 0.4–15 μM)	[30,39,50]
**5**		A1	S5D	*Y. gloriosa*		[32]
**6**	Desmettianoside C	A1	S5E	*Y. desmetiana*	Cytotoxic (HCT116, MCF7, A549, HepG2 IC_50_ = 2.4, 2.6, 10.2, 1.1 μM)	[34]
**7**		A1	S5F	*Y. gloriosa*		[29]
**8**	Yuccaloeside C	A1	S6A	*Y. gloriosa*	Cytotoxic (HeLa IC_50_ = 4.8 μM, HSC-2 y HGF LD_50_ = 1.0, 3.1 μM y L1210 EC_50_ = 0.1 μM) Antifungal (Various strings, MIC 0.39→100 μM)	[7,31,32,49,51,52]
**9**		A1	S6B	*Y. gloriosa*		[29]
**10**	Yuccaloeside E	A1	S6C	*Y. gloriosa*		[7,29]
**11**		A2	Glc	*Y. schidigera*	Cytotoxic (HO-8910, L1210, SW620 IC_50_ = 24.83, 12.33, >100 μM) Antifungal (Inactive)	[28,53,54]
**12**	YS-II/(25R) Timosaponin AIII	A2	S2A	*Y. gloriosa*, *glauca*, *elephantipes*, *desmetiana*	Cytotoxic (HCT116, MCF7, A549, HL60 IC_50_ = 4.9, 4.0, 8.4–16.5, 3.1 μM) Antifungal (*C. albicans* y *C. neoformans* IC_50_ = 5.0, 6.0 μM)	[6,11,34,35,36]
**13**	Elephanoside A	A2	S2B	*Y. elephantipes*	Antifungal (inactive)	[36]
**14**	YS-I/Schidigera-saponin D5	A2	S2D	*Y. gloriosa*	Cytotoxic (SW620 IC_50_ = 63.37 μM) Antifungal * (Various Strings MIC 3.13→100 μM)	[6,14,28,35]
**15**	YS-IV/Schidigera-saponin D4	A2	S3A	*Y. gloriosa*, *schidigera*, *elephantipes*	Antifungal * (Various Strings MIC 3.13–50 μM y *C. neoformans* y *C. albicans* IC_50_ = 15 μM)	[14,35,36]
**16**	Saponin YSM2/Schidigera-saponin D2	A2	S3B	*Y. schidigera*, *glauca*	Antifungal * (Various Strings MIC 1.56→100 μM) Cytotoxic (HL60, A549 IC_50_ = 4.2, 5.9 µM)	[11,14]
**17**	YS-III	A2	S3C	*Y. gloriosa*, *schidigera*	Antifungal (Various strings MIC 3.13–6.25 μM)	[14,35]
**18**	Saponin YSM4/Schidigera-saponin D1	A2	S3D	*Y. schidigera*	Cytotoxic (SW620 IC_50_ = 69.17 μM) Antifungal * (Various strings MIC 3.13→100 μM)	[14,28]
**19**	Asparagoside A	A3	Glc	*Y. schidigera*	Cytotoxic (SW620 IC_50_ = 60.26 μM) Cytotoxic (SW620 IC_50_ = 60.26 μM) Growth Hormone releasing effect (10% release of GH) Anti-alzheimer (IC_50_ = 6.0 μM)	[28,55,56,57]
**20**	Timosaponin AIII	A3	S2A	*Y. macrocarpa*, *gloriosa*	See Section 3.3.2	[15,35,37,55,58,59,60,61,62,63,64,65,66,67,68,69,70]
**21**	Schidigera-saponin D5	A3	S2D	*Y. schidigera*	Cytotoxic (SW620 IC_50_ = 33.91 μM) Antifungal * (Various strings, MIC 3.13→100 μM)	[14,16,28]
**22**	Saponin YSM3/Schidigera-saponin D4	A3	S3A	*Y.schidigera*	Antifungal * (Various strings, MIC 3.13–50 μM)	[14]
**23**	Saponin YSM2/Schidigera-saponin D2	A3	S3B	*Y. schidigera*	Antifungal * (Various strings, MIC 3.13→100 μM)	[14]
**24**		A3	S3C	*Y. schidigera*	Antifungal * (Various strings, MIC 3.13→100 μM)	[14]
**25**	Saponin YSM4/Schidigera-saponin D1	A3	S3D	*Y. schidigera*	Cytotoxic (SW620 IC_50_ = 12.02 μM) Antifungal * (Various strings, MIC 3.13→100 μM)	[14,16,28]
**26**	Yuccoside E	A3	S4D	*Y. filamentosa*		[18]
**27**	Elephanoside G	A4	S3E	*Y. elephantipes*		[19]
**28**	Yucca spirostanoside A_1_	A5	Glc	*Y. schidigera*		[20]
**29**	25(27)-ene-Timosaponin AIII	A5	S2A	*Y. glauca*	Cytotoxic (Various cell lines IC_50_ = 2.5–13.14 μM)	[11,65]
**30**	Yucca spirostanoside A_2_	A5	S2E	*Y. schidigera*		[20]
**31**	Saponin YSM1/Schidigera-saponin A2	A5	S3B	*Y. schidigera*, *glauca*	Cytotoxic (HL60, A549 IC_50_ = 4.9, 6 μM) Antifungal (Various strings, MIC 3.13→100 μM)	[11,14]
**32**	Schidigera-saponin A3	A5	S3C	*Y. schidigera*	Antifungal (Various strings, MIC 3.13→100 μM)	[14,20]
**33**	Schidigera-saponin A1	A5	S3D	*Y. schidigera*	Antifungal (Various strings MIC 3.13–12.5 μM)	[14,20]
**34**		A6	S2A	*Y. gloriosa*		[29]
**35**	Desmettianoside B	A6	S4A	*Y. desmetiana*	Cytotoxic (Various cell lines IC_50_ 1.76–25.07 μM) Anti-inflammatory (Inactive) Molluscicide (LC_100_ = 11 mg/L)	[12,71,72]
**36**	Uttroside B	A6	S4B	*Y. gloriosa*	Cytotoxic (Various strings, IC_50_ 0.5–18.83 μM) Anti-inflammatory (inactive) Platelet aggregation (Inhibition 15.6%)	[29,42,46,50,72,73]
**37**	Desmettianoside A	A6	S5A	*Y. desmetiana*	Molluscicide (LC_100_ = 6 mg/L)	[12]
**38**		A6	S5B	*Y. gloriosa*		[29]
**39**		A6	S5D	*Y. gloriosa*		[29]
**40**	Furcreafurostatin	A6	S6A	*Y. gloriosa*		[29]
**41**		A6	S7A	*Y. gloriosa*		[29]
**42**	(25R) Timosaponin BII	A7	S2A	*Y. gloriosa*, *glauca*, *desmetiana*	Cytotoxic (HL60, A549, HCT116, MCF7, HepG2 IC_50_ = 3.7, 7.0, >100, >100, >100 μM).	[11,21,34]
**43**		A7	S2A	*Y. gloriosa*	Cytotoxic (A549, HCT116, MCF7, HepG2 IC_50_ = >100, >100, >100, >100 μM)	[11,21]
**44**		A7	S2C	*Y. glauca*	Cytotoxic (HL60, A549 IC_50_ = 14.3, 20 μM)	[11]
**45**	Disporoside C	A7	S2D	*Y. gloriosa*, *schidigera*		[16,21]
**46**	Elephanoside B	A7	S3A	*Y. elephantipes*	Antifungal (Inactive)	[36]
**47**		A7	S3D	*Y. schidigera*		[16]
**48**		A8	Glc	*Y. elephantipes*		[19]
**49**		A8	S2C	*Y. elephantipes*	Spermicidal (Inactive, >80% motility)	[19,74]
**50**	Timosaponin BII	A9	S2A	*Y. glauca*	See Section 3.3.3	[11,15,75,76,77,78,79,80,81,82,83]
**51**	Macrostemonoside O/Timosaponin L	A10	S2A	*Y. glauca*	Cytotoxic (Various cell lanes, IC_50_ = 4.4–16.34 μM) Platelet aggregation (Inactive)	[11,61,84,85]
**52**	Macrostemonoside F	A11	S2A	*Y. gloriosa*	Platelet aggregation (inhibition, IC_50_ = 0.02 mM) Anti-inflammatory (NO reduced 62.89%)	[6,86,87]
**53**		A11	S2D	*Y. gloriosa*		[6]
**54**	(25R)-*Yucca* spirostanoiside E_1_	A12	Glc	*Y. schidigera*	Cytotoxic (SW620, Inactive)	[28]
**55**	Elephanoside H	A12	S2A	*Y. gloriosa*, *glauca*, *elephantipes*	Cytotoxic (HL60, A549, Inactive)	[6,11,19]
**56**	(25R)-Yucca spirostanoside E_3_	A12	S2D	*Y. schidigera*	Cytotoxic (SW620 IC_50_ = 29.81 μM)	[28]
**57**		A12	S2E	*Y. schidigera*	Cytotoxic (SW620, Inactive)	[28]
**58**	YS-VIII	A12	S3A	*Y. gloriosa*, *elephantipes*	Antifungal (inactive)	[6,23,36]
**59**		A12	S3B	*Y. glauca*	Cytotoxic (HL60, A549, Inactive)	[11]
**60**	YS-VII	A12	S3C	*Y. gloriosa*, *schidigera*		[6,16,23]
**61**	Schidigera-saponin E1	A12	S3D	*Y. schidigera*	Antifungal * (Various strings, inactive or low activity)	[14,16]
**62**	(25S)-Yucca spirostanoside E_1_	A13	Glc	*Y. schidigera*	Cytotoxic (SW620, Inactive)	[28]
**63**	(25S)-Yucca spirostanoside E_3_	A13	S2D	*Y. schidigera*	Cytotoxic (SW620 IC_50_ = 55.90 μM)	[28]
**64**	(25S)-Yucca spirostanoside E_2_	A13	S2E	*Y. schidigera*	Cytotoxic (SW620, Inactive)	[28]
**65**	Schidigera-saponin E1	A13	S3D	*Y. schidigera*	Antifungal* (Various strings, inactive or low activity)	[14]
**66**	Yucca spirostanoside C_1_	A14	Glc	*Y. schidigera*		[20]
**67**	25(27)-Ene-elephanoside H	A14	S2A	*Y. glauca*	Cytotoxic (HL60, A549, Inactive)	[11]
**68**	Yucca spirostanoside C_2_	A14	S2E	*Y. schidigera*		[20]
**69**	Yucca spirostanoside C_3_	A14	S3A	*Y. schidigera*		[20]
**70**		A14	S3B	*Y. glauca*	Cytotoxic (HL60, A549, Inactive)	[11]
**71**		A14	S3C	*Y. gloriosa*, *schidigera*		[6,20]
**72**	Schidigera-saponin B1	A14	S3D	*Y. schidigera*	Antifungal (Various strings, inactive or low activity)	[14]
**73**	Elephanoside D	A15	S2A	*Y. glauca*, *elephantipes*	Cytotoxic (HL60, A549, Inactive) Antifungal (inactive)	[11,36]
**74**	Elephanoside C	A15	S3A	*Y. elephantipes*	Antifungal (inactive)	[36]
**75**		A16	S3D	*Y. schidigera*		[16]
**76**		A17	S4C	*Y. aloifolia*	Molluscicide (LC_100_ = 10 ppm)	[25]
**77**		A17	S5C	*Y. gloriosa*		[30]
**78**		A17	S5D	*Y. gloriosa*	Cytotoxic (HL60 IC_50_ 1.7–6.5 μM) Anti-alzheimer (NO production IC_50_ = 13.16)	[7,30,88,89,90]
**79**		A17	S5G	*Y. aloifolia*		[24]
**80**		A17	S5H	*Y. aloifolia*		[26]
**81**		A17	S6A	*Y. filamentosa*		[27]
**82**	(25R)-2β-Hydroxytimosaponin AIII	A18	S2A	*Y. gloriosa*, *schidigera*, *glauca*	Cytotoxic (HL60, A549 IC_50_ = 11.3, >20 μM) Antifungal * (Various strings, inactive or low activity)	[11,14,35]
**83**	Schidigera-saponin F1	A18	S3B	*Y. schidigera*	Antifungal * (Various strings, inactive or low activity)	[14]
**84**	Timosaponin AII/Schidigera-saponin F2	A19	S2A	*Y. schidigera*	Antifungal * (Various strings, inactive or low activity)	[14,16]
**85**	Schidigera-saponin F1	A19	S3B	*Y. schidigera*	Antifungal * (Various strings, inactive or low activity)	[14]
**86**	25(27)-Ene-2β-hydroxytimosaponin AIII/Schidigera-saponin C2	A20	S2A	*Y. schidigera*, *glauca*	Cytotoxic (HL60, A549 IC50 = 5.0, >20 μM) Antifungal (Various strings, inactive or low activity)	[11,14,20]
**87**	Schidigera-saponin C1	A20	S3B	*Y. schidigera*	Antifungal (Various strings, inactive or low activity)	[14,20]
**88**	YG4	A21	S5C	*Y. gloriosa*		[30]
**89**		A22	S2A	*Y. glauca*	Cytotoxic (HL60, A549 IC50 = 17.8, >20 μM)	[11]
**90**	Timosaponin N	A23	S2A	*Y. glauca*	Cytotoxic (HL60, A549 IC_50_ 9.2, >20 μM)	[11]
**91**	25(27)-Ene-Macrostemonoside J	A24	S2A	*Y. glauca*	Cytotoxic (HL60, A549 IC50 = 13.3, >20 μM)	[11]
**92**	(25R)-12β-Hydroxytimosaponin AIII/YS-XIII	A25	S2A	*Y. gloriosa*, *glauca*	Cytotoxic (HL60, A549, Inactive)	[11,22]
**93**		A25	S2D	*Y. gloriosa*		[6]
**94**		A25	S3C	*Y. gloriosa*		[22]
**95**	Yucca spirostanoside B_1_	A26	Glc	*Y. schidigera*		[20]
**96**	Yucca spirostanoside B_2_	A26	S2E	*Y. schidigera*		[20]
**97**	Yucca spirostanoside B_3c_	A26	S3C	*Y. schidigera*		[20]
**98**	Elephanoside E	A27	S2A	*Y. elephantipes*	Antifungal (inactive)	[36]
**99**	Elephanoside F	A27	S3A	*Y. elephantipes*	Antifungal (inactive)	[36]
**100**	YS-IX	A28	S4B	*Y. gloriosa*	Cytotoxic (HL60 IC_50_ = 7.2 μM)	[23,89]
**101**	YS-X	A28	S5D	*Y. gloriosa*	Cytotoxic (HL60, 40% growth inhibition at 10 μM)	[23,89]
**102**	YS-VI	A29	S2A	*Y. gloriosa*		[23]
**103**		A30	S3B	*Y. schidigera*		[20]
**104**		A31	S3B	*Y. glauca*	Cytotoxic (HL60, A549, inactive)	[11]
**105**		A32	S2A	*Y. gloriosa*		[22]
**106**	Yuccalan	A33	S2F	*Y. smalliana*	Antifungal (*Fusarium oxysporum* and *Rhizoctonia solani*, low activity)	[33]

* Saponins or bioactivity described in epimers mixture.

**Table 2:**
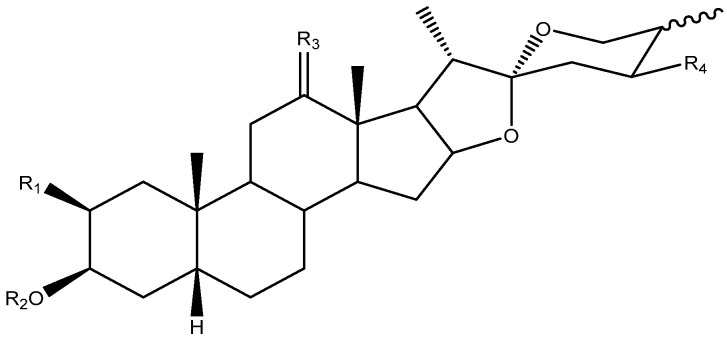
Bioactivity of spirostanic saponins by *Y. glauca* and by cisplatin and etoposide as control substances [11].

Compound	R_1_	R_2_	R_3_	R_4_	C-25	IC_50_ HL-60 (µM)	IC_50_ A549 (µM)
**29**	H	S2A	H, H	H	Δ^25(27)^	2.5 ± 0.47	7.3 ± 0.63
**67**	H	S2A	O	H	Δ^25(27)^	>20	
**70**	H	S3B	O	H	Δ^25(27)^	>20	
**59**	H	S3B	O	H	25R	>20	
**104**	H	S3B	O	OH	25S	>20	
**12**	H	S2A	H, H	H	25R	3.1 ± 0.35	8.4 ± 0.34
**92**	H	S2A	β-OH	H	25R	>20	
**55**	H	S2A	O	H	25R	>20	
**86**	OH	S2A	H, H	H	Δ^25(27)^	5.0 ± 0.09	>20
**82**	OH	S2A	H, H	H	25R	11.3 ± 1.42	>20
**31**	H	S3B	H, H	H	Δ^25(27)^	4.9 ± 0.43	6.0 ± 0.65
**16**	H	S3B	H, H	H	25R	4.2 ± 0.37	5.9 ± 0.43
Cisplatin						1.7 ± 0.06	2.1 ± 1.10
Etoposide						0.39 ± 0.08	

**Table 3:**
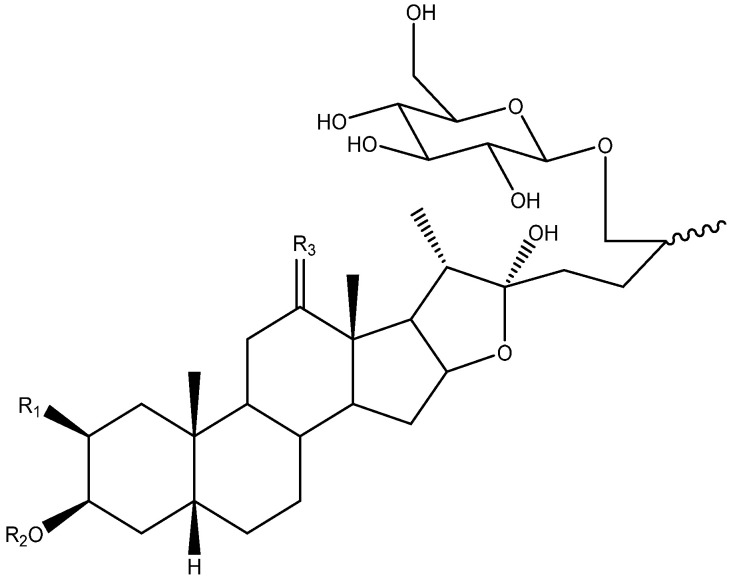
Bioactivity of furostanic saponins by *Y. glauca* and by Cisplatin and Etoposide as control substances [11].

Compound	R_1_	R_2_	R_3_	C-25	IC_50_ HL-60 (µM)	IC_50_ A549 (µM)
**91**	OH	S2A	H, H	Δ^25(27)^	13.3 ± 0.09	>20
**89**	OH	S2A	H, H	25R	17.8 ± 2.47	>20
**90**	OH	S2A	H, H	25S	9.2 ± 1.21	>20
**51**	H	S2A	H, H	Δ^25(27)^	4.4 ± 0.10	11.9 ± 0.70
**42**	H	S2A	H, H	25R	3.7 ± 0.55	7.0 ± 0.29
**50**	H	S2A	H, H	25S	3.3 ± 0.15	9.3 ± 2.07
**73**	H	S2A	O	25R	>20	
**44**	H	S2C	H, H	25R	14.3 ± 0.07	>20
Cisplatin					1.7 ± 0.06	2.1 ± 1.10
Etoposide					0.39 ± 0.08	-

**Table 4:**
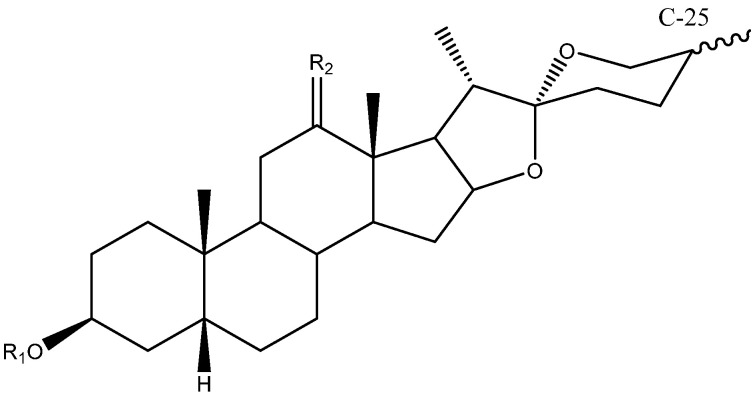
Cytotoxic activity of the saponins in *Y. schidigera* [28].

Compound	R_1_	R_2_	C-25	IC_50_ (µM)
Control (5-Fluorouracil)				10.00 ± 0.15
**54**	Glc	O	R	>100
**62**	Glc	O	S	>100
**57**	S2E	O	R	>100
**64**	S2E	O	S	>100
**56**	S2D	O	R	29.81 ± 0.21
**63**	S2D	O	S	55.90 ± 0.78
**11**	Glc	H, H	R	>100
**19**	Glc	H, H	S	60.26 ± 4.53
**14**	S2D	H, H	R	63.37 ± 0.70
**21**	S2D	H, H	S	33.91 ± 1.27
**18**	S3D	H, H	R	69.17 ± 1.24
**25**	S3D	H, H	S	12.02 ± 1.43

**Table 5:**
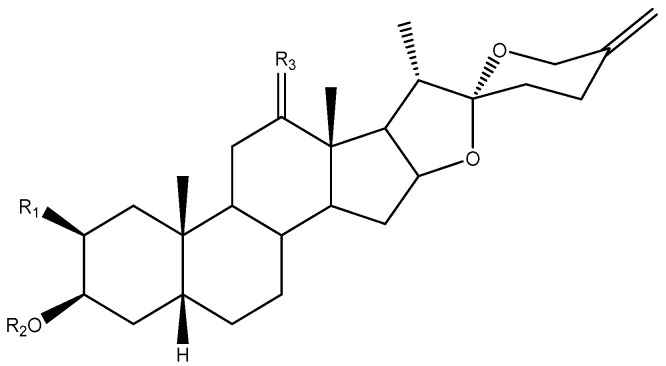
MIC (µM) values calculated for the individual saponins from *Y. schidigera* [14].

Compound	R_1_	R_2_	R_3_	Sc	Ca	Ha	Pn	Ka	Dh
**33**	H	S3D	H_2_	3.13	6.25	3.13	3.13	12.5	6.25
**31**	H	S3B	H_2_	12.5	12.5	3.13	3.13	>100	>100
**32**	H	S3C	H_2_	12.5	12.5	6.13	3.13	>100	>100
**72**	H	S3D	O	>100	>100	>100	>100	>100	>100
**87**	OH	S3B	H_2_	100	100	>100	100	>100	>100
**86**	OH	S2A	H_2_	>100	>100	>100	>100	>100	>100

**Table 6:**
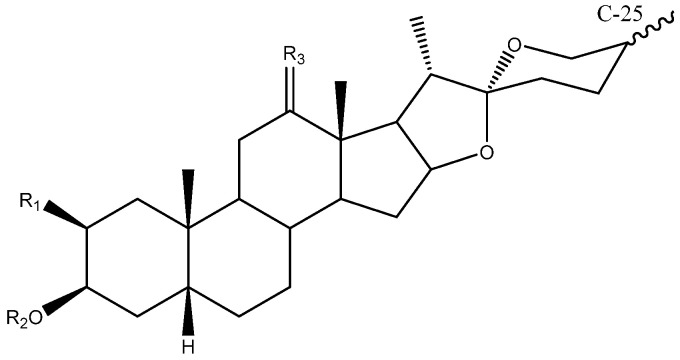
Calculated MIC (µM) values for the epimer pairs on the C-25 position in *Y. schidigera* [14].

Compounds	R_1_	R_2_	R_3_	Sc	Ca	Ha	Pn	Ka	Dh
**18 + 25**	H	S3D	H_2_	6.25	50	3.13	3.13	>100	6.25
**16 + 23**	H	S3B	H_2_	25	>100	3.13	12.5	>100	50
**24 + 17**	H	S3C	H_2_	6.25	>100	1.56	3.13	>100	6.25
**22 + 15**	H	S3A	H_2_	12.5	25	3.13	6.25	50	6.25
**21 + 14**	H	S2D	H_2_	12.5	12.5	6.25	3.13	>100	>100
**61 + 65**	H	S3D	O	100	>100	100	>100	>100	>100
**83 + 85**	OH	S3B	H_2_	100	>100	>100	>100	>100	100
**84 + 82**	OH	S2A	H_2_	>100	>100	>100	100	>100	>100

**Table 7:** Timosaponin AIII (**20**) bioactivities.

Activity	Description	Value	Value Range	Refs.
Cytotoxic	Reduces the viability of cancer cells.	IC_50_/EC_50_	0.35–22.1 µM/0.02–5.12 µM	[38,58,65,66,67,68,70,79]
Anti-inflammatory	Can moderate the physiological response to inflammation.	IC_50_/% BALF recruitment	1.21, 1.82 µM/64% lower (50mg/kg)	[15,59]
Effect on platelet aggregation	Inhibition or induction to platelet aggregation.	IC_50_/% inhibition	4.36 µM/80–90% (2–50 µM)	[60,61]
Anti-Alzheimer	Prevents and improves the condition of Alzheimer’s patients.	IC_50_/% Aβ_42_ reduction	2.3–7.45 µM/42% (5 µM)	[55,63]
Anti-diabetic osteoporosis	Prevents and improves the condition of patients with diabetes-related osteoporosis.	Better anti-AGEs and anti-osteoporosis effects	0.1 µM	[64]

**Table 8:** Timosaponin BII (**50**) bioactivities.

Activity	Description	Value	Value Range	Refs.
Cytotoxic	Reduces the viability of cancer cells.	IC_50_/% tumor growth inhibition	3.3, 9.3 µM/65–84% (10–15% wt)	[11,76]
Anti-inflammatory	Can moderate the physiological response to inflammation.	IC_50_/% damaged cells viability increment.	0.77, 1.57 µM/61.5–74.8% (5–50 µM)	[15,78]
Effect on platelet aggregation	Inhibition or induction to platelet aggregation.	Concentration to inhibit ADP-induced aggregation	50–100 µM	[79,103]
Anti-Alzheimer	Prevents and improves the condition of Alzheimer’s patients.	Inhibition of IL6, IL1β and TNFα expressions.	0.313–5 mg/mL	[75]
Anti-diabetic	Prevents and improves the condition of diabetic patients.	α-glucosidase inhibition		[104]
Cardioprotective	Preventive and reparative capacity against cardiac disorders.	Reversal of ISO-induced disorders/overall improvement of the conditions	50 mg/kg–100 mg/kg/100–200 µM	[80,82]
Antiapoptotic	Protecting HUVECs against high-glucose- induced apoptosis.			[99]
